# Supplementary figures and images for: GWAS and functional studies suggest a role for altered DNA repair in the evolution of drug resistance in Mycobacterium tuberculosis
Source: eLife. 2023 Jan 25;12:e75860. doi: 10.7554/eLife.75860 (PMC9876569; doi:10.7554/eLife.75860)

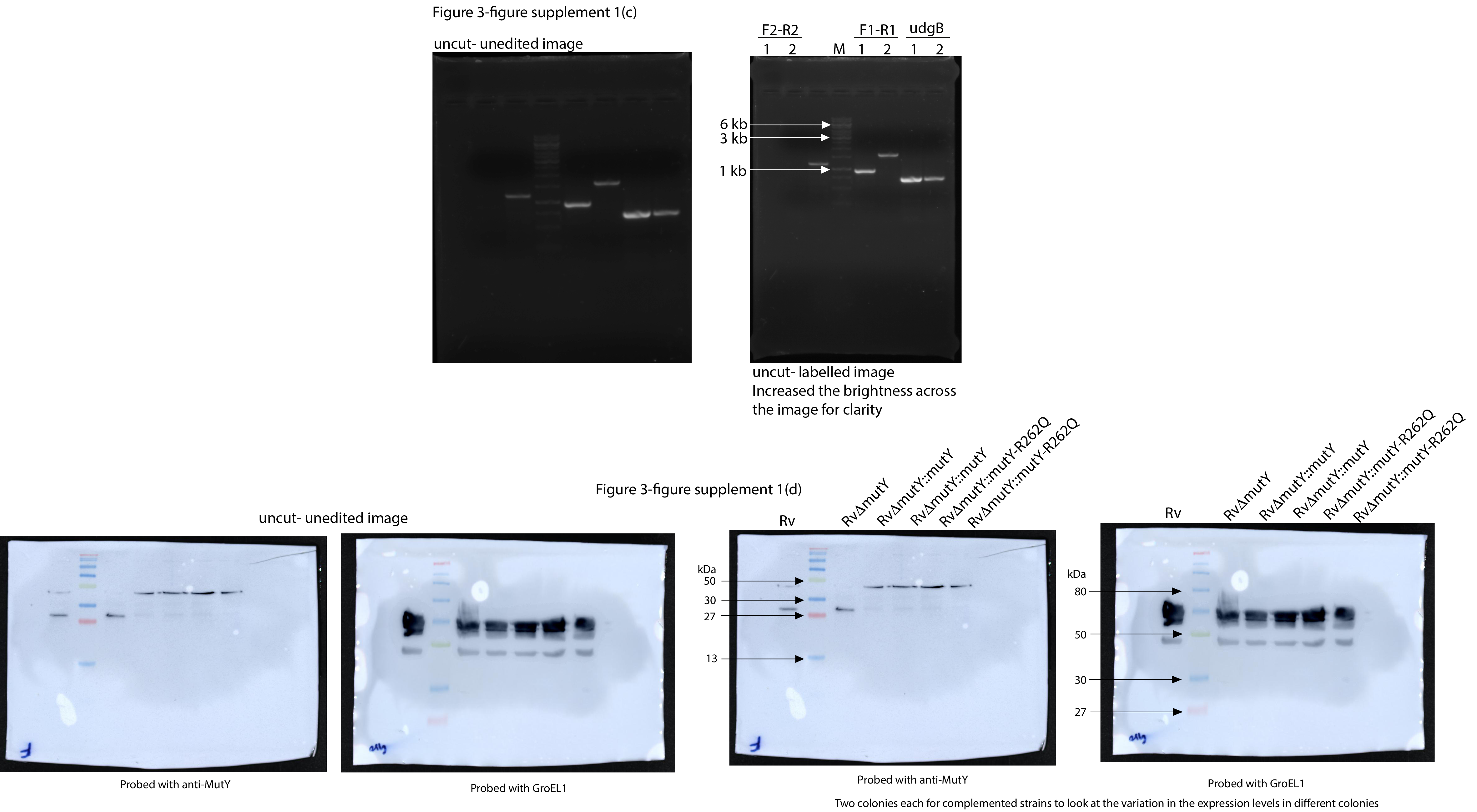

Supplement: Figure 3—figure supplement 1—source data 1. [file elife-75860-fig3-figsupp1-data1.zip › Figure3-FigureSupplement 1-SourceData-1 (6).tif]

Figure 8b- Raw Data

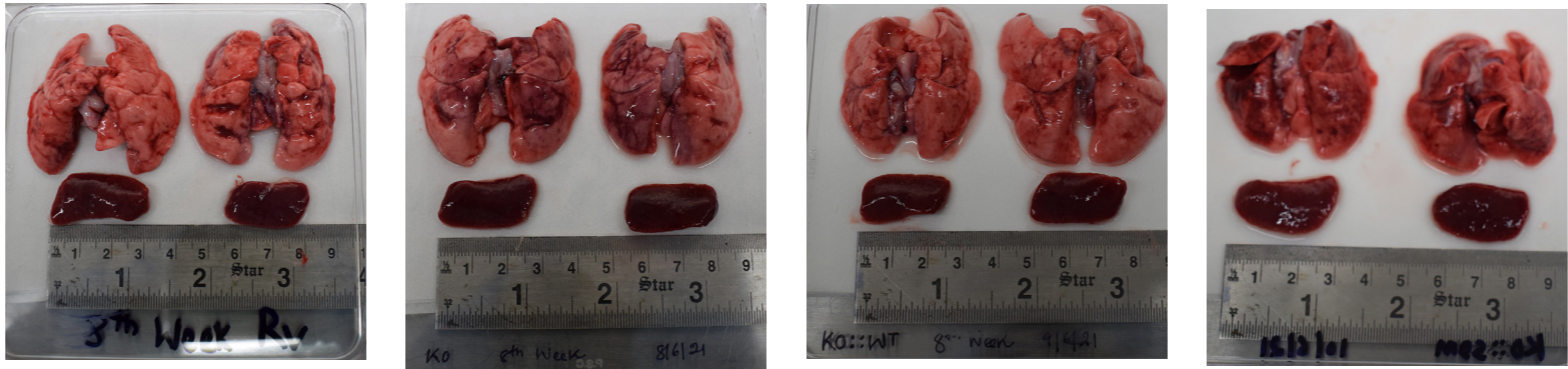

Supplement: Figure 8—source data 1. [file elife-75860-fig8-data1.pdf]

Figure 8c- Raw Data

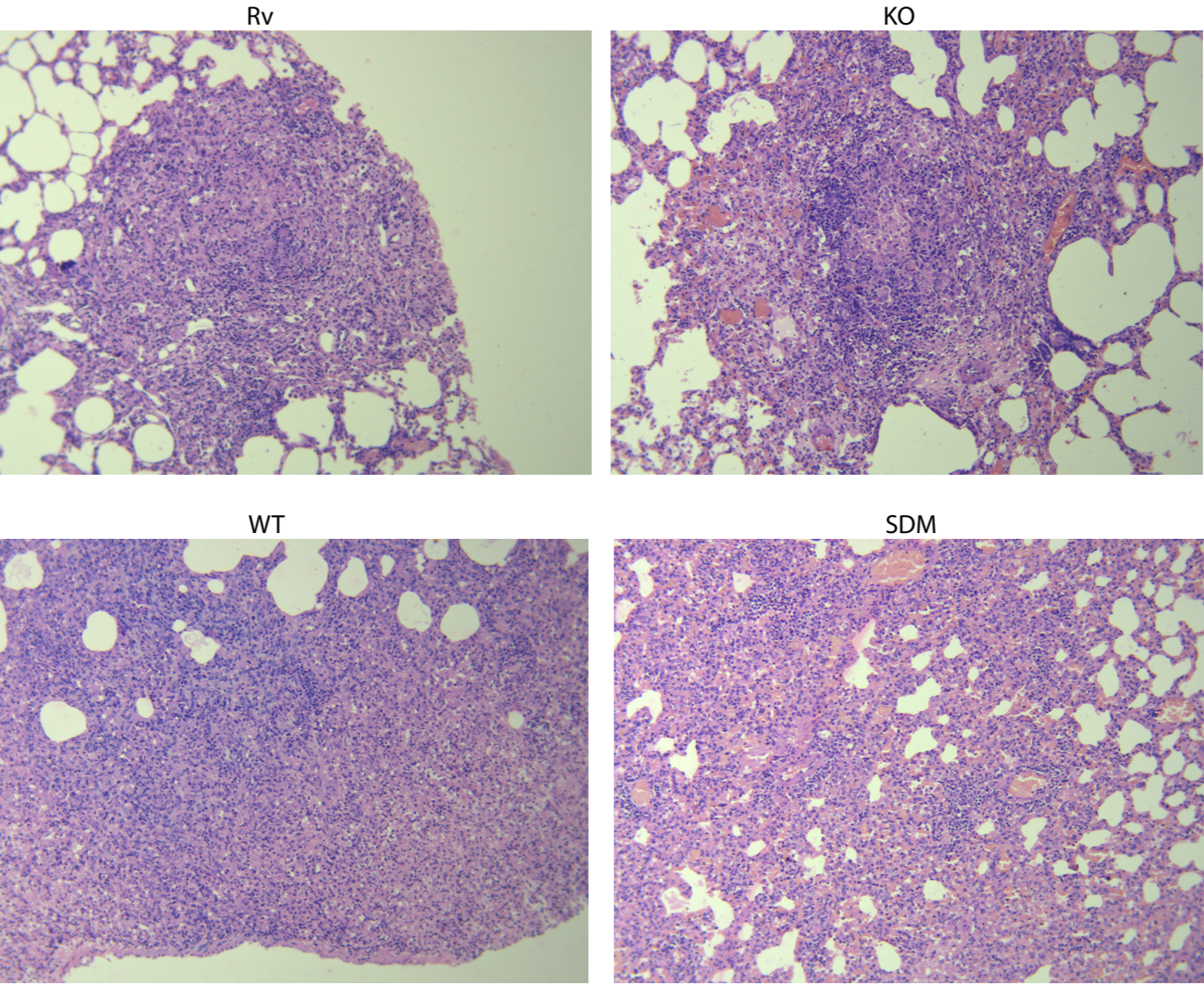

Supplement: Figure 8—source data 2. [file elife-75860-fig8-data2.pdf]

Figure 8-figure supplement 1-Raw Data

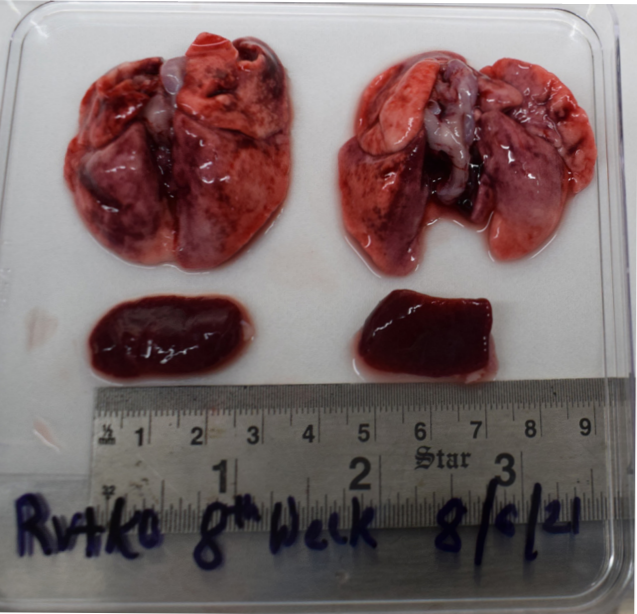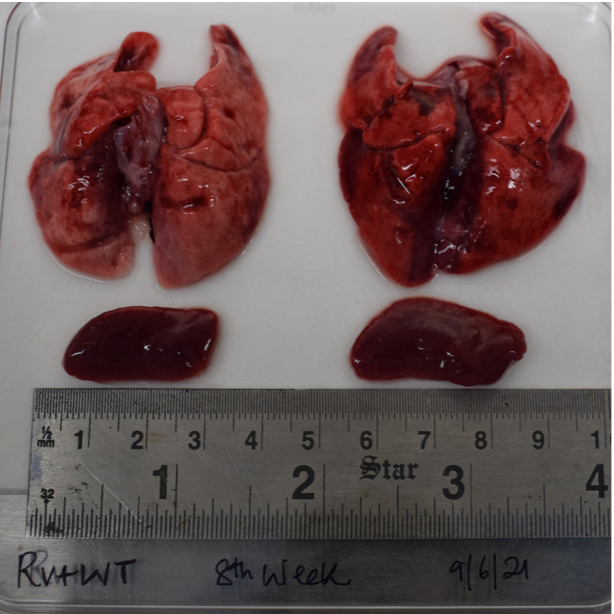

Rv+SDM

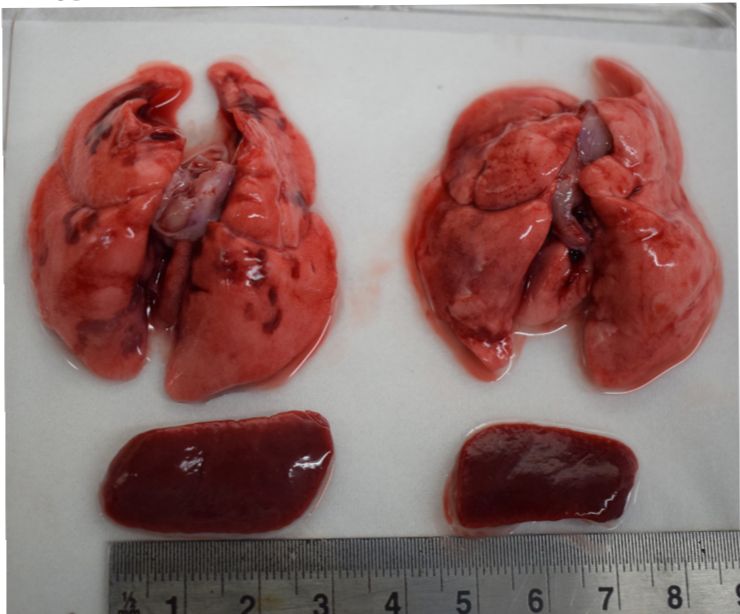

Supplement: Figure 8—figure supplement 1—source data 1. [file elife-75860-fig8-figsupp1-data1.pdf]
