# Supplementary material for: GWAS and functional studies suggest a role for altered DNA repair in the evolution of drug resistance in Mycobacterium tuberculosis
Source: eLife. 2023 Jan 25;12:e75860. doi: 10.7554/eLife.75860 (PMC9876569; doi:10.7554/eLife.75860)
Supplement: Transparent reporting form [file elife-75860-transrepform1.pdf]

Westbrook Centre
Milton Road
Cambridge CB4 1YG, UK
P 01223 855340
W elifesciences.org
T @elife

eLife's transparent reporting form

We encourage authors to provide detailed information *within their submission* to facilitate the interpretation and replication of experiments. Authors can upload supporting documentation to indicate the use of appropriate reporting guidelines for health-related research (see EQUATOR Network), life science research (see the BioSharing Information Resource), or the ARRIVE guidelines for reporting work involving animal research. Where applicable, authors should refer to any relevant reporting standards documents in this form.

If you have any questions, please consult our Journal Policies and/or contact us: editorial@elifesciences.org.

Sample-size estimation

- You should state whether an appropriate sample size was computed when the study was being designed
- You should state the statistical method of sample size computation and any required assumptions
- If no explicit power analysis was used, you should describe how you decided what sample (replicate) size (number) to use

Please outline where this information can be found within the submission (e.g., sections or figure legends), or explain why this information doesn't apply to your submission:

We have downloaded the 2773 clinical strains of *Mycobacterium tuberculosis*. Based on the phenotype of the strains reported in the previous studies, we divided the strains in drug susceptible and drug resistant. Using 422MDR/XDR strains and the 1815 drug susceptible strains we performed association analysis. All the strains used for the analysis are reported in the Supplementary Tables.

We have performed Whole Genome Sequence analysis of the strains that were passaged *ex vivo* or isolated from the guinea pig lungs 8-week post infection. We have sequenced 24 samples and each sample contains genomic DNA isolated from 10 different colonies.

Replicates

- You should report how often each experiment was performed
- You should include a definition of biological versus technical replication
- The data obtained should be provided and sufficient information should be provided to indicate the number of independent biological and/or technical replicates
- If you encountered any outliers, you should describe how these were handled
- Criteria for exclusion/inclusion of data should be clearly stated
- High-throughput sequence data should be uploaded before submission, with a private link for reviewers provided (these are available from both GEO and ArrayExpress)

Please outline where this information can be found within the submission (e.g., sections or figure legends), or explain why this information doesn't apply to your submission:

eLife Sciences Publications, Ltd is a limited liability non-profit non-stock corporation incorporated in the State of Delaware, USA, with company number 5030732, and is registered in the UK with company number FC030576 and branch number BR015634 at the address Westbrook Centre, Milton Road, Cambridge CB4 1YG.

- We performed at least two independent experiments with biological triplicates.
- We have reported data from one independent experiment consisting of at least biological triplicates.
- For mutation rate analysis, we have performed experiment using six independent colonies. These points are mentioned in the methods and legends of the revised manuscript.
- Biological replication means the experiments two independent set of experiments. One set of independent experiment consists of at least biological triplicates
- Replication of each experiment is mentioned both in methods and figure legend section.
- We have included all the data points in the analysis.

eLife Sciences Publications, Ltd is a limited liability non-profit non-stock corporation incorporated in the State of Delaware, USA, with company number 5030732, and is registered in the UK with company number FC030576 and branch number BR015634 at the address Westbrook Centre, Milton Road, Cambridge CB4 1YG.

Statistical reporting

- Statistical analysis methods should be described and justified
- Raw data should be presented in figures whenever informative to do so (typically when N per group is less than 10)
- For each experiment, you should identify the statistical tests used, exact values of N, definitions of center, methods of multiple test correction, and dispersion and precision measures (e.g., mean, median, SD, SEM, confidence intervals; and, for the major substantive results, a measure of effect size (e.g., Pearson's r, Cohen's d)
- Report exact p-values wherever possible alongside the summary statistics and 95% confidence intervals. These should be reported for all key questions and not only when the p-value is less than 0.05.

Please outline where this information can be found within the submission (e.g., sections or figure legends), or explain why this information doesn't apply to your submission:

- We have performed ANOVA and students t-test analysis using Graphpad Prism software.
- Statistical analysis is mentioned in the methods and figure legends.
- We have provided the p-values for each analysis. These are given in supplementary Tables file and in the figure legends.

(For large datasets, or papers with a very large number of statistical tests, you may upload a single table file with tests, Ns, etc., with reference to sections in the manuscript.)

Group allocation

- Indicate how samples were allocated into experimental groups (in the case of clinical studies, please specify allocation to treatment method); if randomization was used, please also state if restricted randomization was applied
- Indicate if masking was used during group allocation, data collection and/or data analysis

Please outline where this information can be found within the submission (e.g., sections or figure legends), or explain why this information doesn't apply to your submission:

Clinical strains were downloaded from the NCBI or EBI (mentioned in the manuscript). These clinical strains were divided in the drug susceptible and drug resistant strains. Phenotypic data was obtained from the previously published studies. The reference for each study is provided in the manuscript file.

Raw data of sequenced strains is deposited to NCBI under Bioproject PRJNA885615.

Additional data files ("source data")

- We encourage you to upload relevant additional data files, such as numerical data that are represented as a graph in a figure, or as a summary table
- Where provided, these should be in the most useful format, and they can be uploaded as "Source data" files linked to a main figure or table
- Include model definition files including the full list of parameters used
- Include code used for data analysis (e.g., R, MatLab)
- Avoid stating that data files are "available upon request"

eLife Sciences Publications, Ltd is a limited liability non-profit non-stock corporation incorporated in the State of Delaware, USA, with company number 5030732, and is registered in the UK with company number FC030576 and branch number BR015634 at the address Westbrook Centre, Milton Road, Cambridge CB4 1YG.

Please indicate the figures or tables for which source data files have been provided:

Source data file for Figures - 2, 3, 4, 5, 7, 8, Figure 3-figure supplement 1, Figure 5-figure supplement 1 is provided.<br>Raw data images for the Figures- Figure 8-figure supplement 1, Figure 8b, Figure 8c, Figure 3-figure supplement 1c, Figure 3-figure supplement 1d is provided.

eLife Sciences Publications, Ltd is a limited liability non-profit non-stock corporation incorporated in the State of Delaware, USA, with company number 5030732, and is registered in the UK with company number FC030576 and branch number BR015634 at the address Westbrook Centre, Milton Road, Cambridge CB4 1YG.
